# Flux balance impact degree: a new definition of impact degree to properly treat reversible reactions in metabolic networks

**DOI:** 10.1093/bioinformatics/btt364

**Published:** 2013-07-10

**Authors:** Yang Zhao, Takeyuki Tamura, Tatsuya Akutsu, Jean-Philippe Vert

**Affiliations:** ^1^Bioinformatics Center, Kyoto University, Kyoto, Japan, ^2^Centre for Computational Biology, Mines ParisTech, 35 rue Saint-Honoré, 77305 Fontainebleau, France and ^3^Institut Curie and ^4^INSERM U900, Paris, France

## Abstract

**Motivation:** Metabolic pathways are complex systems of chemical reactions taking place in every living cell to degrade substrates and synthesize molecules needed for life. Modeling the robustness of these networks with respect to the dysfunction of one or several reactions is important to understand the basic principles of biological network organization, and to identify new drug targets. While several approaches have been proposed for that purpose, they are computationally too intensive to analyze large networks, and do not properly handle reversible reactions.

**Results:** We propose a new model**—**the flux balance impact degree**—**to model the robustness of large metabolic networks with respect to gene knock-out. We formulate the computation of the impact of one or several reaction blocking as linear programs, and propose efficient strategies to solve them. We show that the proposed method better predicts the phenotypic impact of single gene deletions on *Escherichia coli* than existing methods.

**Availability:** https://sunflower.kuicr.kyoto-u.ac.jp/∼tyoyo/fbid/index.html

**Contact:** takutsu@kuicr.kyoto-u.ac.jp or Jean-Philippe.Vert@mines.org

**Supplementary information:** Supplementary data are available at *Bioinformatics* online.

## 1 INTRODUCTION

Metabolic pathways are complex systems of biochemical reactions taking place in every living cell to degrade substrates and synthesize molecules needed for life. Any metabolic dysfunction may lead to the impossibility to degrade or produce crucial molecules for the organism, potentially inducing disease or death. Yet cells seem to be able to maintain their normal functions despite many perturbations, such as the gene knock-out or DNA mutations perturbing the functions of proteins, while being sensitive to some specific attacks (Jeong *et al.*, 2001). Understanding and modeling the organizational principles underlying the robustness of metabolic networks with respect to gene perturbations is important not only to shed light on basic principles of life, but also to identify weaknesses that may lead to new drug targets to kill pathogens or cancer cells (Behre *et al.*, 2008).

Conceptually, a metabolic network can be considered as a network consisting of metabolites and enzyme (gene)-catalyzed reactions that bridge these metabolites to transformation processes. A gene perturbation, such as knock-out or DNA mutation, can inhibit one or several reactions in a metabolic system. The impact of this perturbation on the cell phenotype can vary widely, ranging from no effect to cell death, depending on how many other reactions and crucial metabolites are impacted in cascade.

Several approaches have been proposed to model and predict the phenotypic impact of inhibiting one or several genes through metabolic network perturbation. Flux balance analysis (FBA) is a constraint-based mathematical model, which uses the stoichiometry of a given metabolic network along with a biologically relevant objective function to identify optimal reaction flux distributions (Raman and Chandra, 2009; Varma and Palsson, 1994). It can be used to predict the effect of inhibiting one or several reactions by assessing how the objective function changes after the perturbation (Edwards and Palsson, 2000b). A related approach proposed by Segre *et al.* (2002) is the method of minimization of metabolic adjustment (MOMA), which predicts the flux vectors of gene knock-out mutants by imposing the constraint that mutants operate by minimizing their metabolic adjustment with respect to the wildtype. Flux variability analysis (FVA) assesses the range of possible fluxes for each reaction when the system runs near optimality, and has been used to evaluate the consequences of metabolic perturbation (Shlomi *et al.*, 2009); however, FVA has not been used, to our knowledge, to predict metabolic gene essentiality. A limitation of FBA, MOMA and FVA is the difficulty to define a relevant objective function: for example, the objective function to predict cell growth typically involves a linear combination of more than 100 metabolites (Raman and Chandra, 2009).

**Fig. 1.**
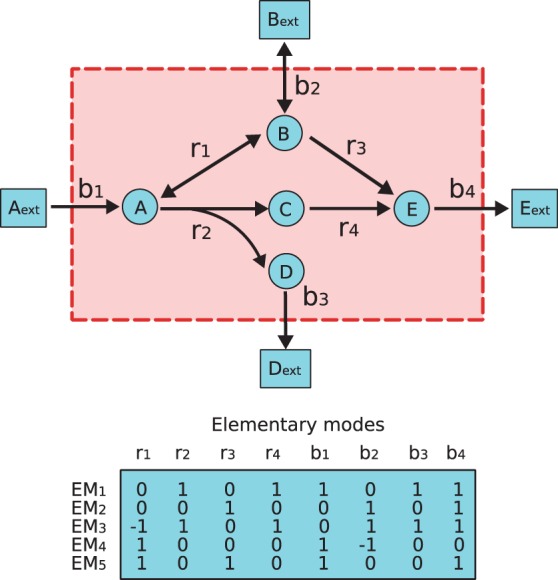
The EMs of an example network, where A, B, C, D and E (cycles) are given as internal metabolites that need to fulfill a steady-state, while Aext, Bext and Eext (squares) are given as external metabolites that need not be balanced in this scheme. Double-headed arrows labeled as *r*_1_ and *b*_2_ represent reversible reactions. Unfilled arrows labeled as 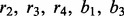 and *b*_4_ represent irreversible reactions. EMs of this example are given in the table, where each row represents an EM in which value 0 means that the corresponding reactions are not included in this EM (See also a metatool format of this example in Supplementary Materials, which can be directly used for open software.)

Other approaches model the effect of gene knock-out using the concept of elementary modes (EMs), which are minimal sets of reactions that can operate at the steady state, such that all irreversible reactions involved are used in the appropriate direction (Schuster and Hilgetag, 1994; Schuster *et al.*, 2000). Figure 1 shows, for example, the EMs of a simple network. With elementary mode analysis (EMA), Stelling *et al.* (2002) proposed that the viability of a mutant carrying mutation in a single gene can be predicted by the number of EMs that do not require the gene, a concept that has been generalized to define a notion of network robustness (Behre *et al.*, 2008; Wilhelm *et al.*, 2004). EM-based methods, however, suffer from computational cost. Although several tools exist to compute EMs of middle-size networks (Klamt *et al.*, 2007; Trinh *et al.*, 2009), they do not scale to large networks because the number of EMs grows exponentially with the network size (Acuna *et al.*, 2009; Haus *et al.*, 2008; Klamt and Stelling, 2002). Acuna *et al.* (2009) proved that counting the number of EMs is # *P-complete*, and although Haus *et al.* (2008) proposed an efficient method for computing EMs, it is still not polynomial.

Alternatively, Klamt and Gilles (2004) proposed a model of minimal cut set (MCS) as a minimal set of reactions in metabolic networks whose disturbances cause dysfunction. However, their computation of the MCSs is based on EMs, which becomes infeasible to analyze large-scale metabolic networks. A method based on a dual framework was recently proposed by Ballerstein *et al.* (2012), which can determine MCSs without calculating the EMs; the formulation is, however, also not scalable for large networks. Finally, different from many other approaches, the concept of synthetic accessibility (SA), proposed by Wunderlich and Mirny (2006), predicts the viability of mutant strains from the network topology, without knowledge of stoichiometry or biomass growth, but with specification of medium inputs and biomass outputs.

An alternative route to model the impact of a perturbation on a metabolic network is to start from a dynamic model of metabolism and assess how the model is impacted when a reaction is inhibited. Boolean models, in particular, are popular to describe and analyze large-scale metabolic networks (Handorf *et al.*, 2008; Sridhar *et al.*, 2008; Tamura *et al.*, 2010). Concepts of damage (Lemke *et al.*, 2004; Smart *et al.*, 2008) and topological impact degree (Jiang *et al.*, 2009) were extensively studied in recent years, where Lemke *et al.* (2004); Smart *et al.* (2008) and Jiang *et al.* (2009) define the impact of a reaction as the number of reactions inactivated by an iterative procedure, mimicking a cascade of failures. Tamura *et al.* (2011) borrowed the concept of topological impact degree of Jiang *et al.* (2009) and extended it to deal with cycles in metabolic networks. However, these methods can not properly handle reversible reactions.

In this study, we propose a new model to assess the impact of gene perturbations on a metabolic network, together with efficient algorithms to compute it of large-scale networks. The model, which we call *flux balance impact degree* (FBID), builds on the concept of steady-state fluxes and variability of FBA and FVA. The FBID of a perturbation is defined as the number of reactions that become inactive in all steady states after perturbation. We show that the FBID can be computed either by enumerating all EMs of the metabolic network, or by solving a series of linear programs, the later scaling much better to large networks. In contrast to techniques like FBA, FVA and MOMA, the new FBID does not require the definition of a specific objective function to model growth. Experiments on the *Escherichia coli* metabolic network show that FBID is competitive with existing approaches. It is computationally efficient even for global metabolic networks, where it outperforms existing approaches in terms of prediction accuracy.

## 2 METHODS

### 2.1 Flux balance impact degree

We represent a metabolic network by its 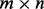 stoichiometric matrix **S**, where *m* is the number of metabolites and *n* is the number of reactions in the network. The activity of the network is represented by a flux vector 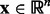, which contains all internal and exchange reactions in the network. A metabolic network for which mass balance constraints are satisfied is assumed to be in steady state, meaning that the flux vector satisfies the following:
(1)
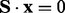



In addition, flux vectors must satisfy additional constraints of the form 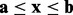, where 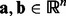 are lower/upper limits for the fluxes in the network, to account to various constraints in the system. In particular, we can use them to encode the reversibility or irreversibility of reactions by setting the value of lower limits 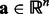 to be –1 for reversible reactions and 0 for irreversible ones, while the upper limits **b** are set to 1. This ensures that fluxes are bounded by [–1, 1] for reversible reactions, and by [0,1] for irreversible ones.

The metabolic networks we consider are usually under-determined because there are usually more reactions than metabolites (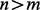). The set of admissible steady-state fluxes of the network is then the convex polytope:
(2)
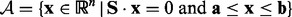

Note that we assume that all reactions can be activated at steady state, meaning that for each reaction 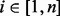 there exists a flux vector **x** in 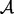 that satisfies 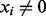. If this is not the case, we just remove the corresponding reactions from the network.

The perturbations we consider lead to gene knock-out, either by drug action or through DNA mutations. In our formalism, we represent a perturbation as a subset 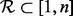 of reactions inhibited by the perturbation. Inhibiting one or several reactions reduces their fluxes to zero in any steady state, and therefore reduces the set of admissible steady-state fluxes (2) to the reduced feasible set:
(3)
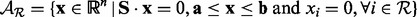

We can now formally define a new notion of FBID of a perturbation, as the number of reactions that are inhibited in any steady-state following the perturbation:

**Definition 1**

*A reaction *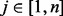 *is impacted by a perturbation *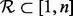 *if *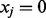 *holds for all *
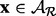
*. The FBID of *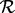 *is the number of reactions impacted by *
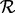
*.*

We note that, by definition, all reactions in 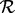 are impacted by 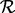, and therefore the FBID of 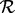 is at least as large as the cardinality of 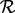 itself. It can be strictly larger when other reactions, not directly in 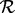, are directly or indirectly affected by the knock-out of 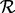. For example, in the network represented on Figure 1, let 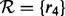. We see that reactions *r*_2_ and *b*_3_ affected by the knock-out of 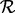, and therefore the FBID of 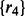 is 3, the total number of inhibited reactions.

### 2.2 EM-based computation

In this section, we show how to compute the FBID of any perturbation 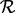 from the enumeration of the EMs of the network. Following Schuster *et al.* (2000), we recall that an EM is a minimal set of reactions that allows a metabolic network to function in a steady state, i.e. a minimal set of reactions 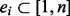 such that there exists a flux vector 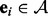 satisfying the condition 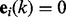 if and only if 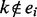, where 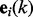 denotes the flux value of reaction *k* in the flux vector 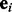. Interestingly, the set *E* of all EMs of a metabolic network forms a basis of admissible steady state fluxes (Schuster *et al.*, 2002).

We now propose an algorithm to compute the FBID of a perturbation 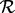 from the list *E* of EMs of a metabolic network:

1. Compute the set of EMs *E* of the given metabolic network.
2. For a perturbation 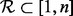, select the subset of EMs from *E* that do not contain reactions in 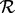, that is:
(4)
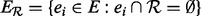

3. The set of reactions 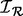 impacted by 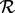 is computed as the set of reactions that are not contained in any EMs of 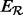:
(5)
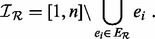

We now prove that this algorithm is correct, in the sense that the set 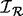 it outputs in (5) is precisely the set of reactions impacted by 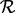 in the sense of Definition 1. Let us first consider a reaction 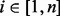 that is not in 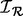. From (5) there exists an EM 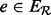 such that 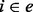. The flux vector 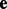 corresponding to *e* is by definition admissible and has zero flux on the perturbed reactions by (4). It therefore belongs to 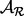, and because it has a non-zero flux on reaction *i*, this reaction is not impacted by 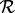 according to Definition 1. This shows that all impacted reactions are in 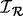. Conversely, let us consider a reaction *i* that is not impacted by 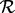 in the sense of Definition 1. This means that there exists a flux 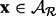 such that 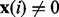. However, by Lemma 1 of Schuster *et al.* (2002), as 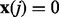 for 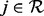 it can be decomposed as a linear combinations of EMs that have themselves zero flux on 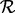, meaning that it can be decomposed as a linear combination of EMs in 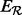. Because 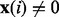, there must be at least an EM in 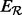 with non-zero flux in *i*, meaning that 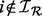. This shows that all reactions in 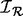 are impacted, which concludes the proof.

To run this algorithm, we need to first compute all EMs of a network, which is a computational demanding task (Gagneur and Klamt, 2004). Although computation of all EMs of a given metabolic network may demand a high computation cost, this operation needs to be performed only once. The rest of the computation (steps 2 and 3) can be done fast. We use the example given in Figure 1 to elucidate the step 2 and 3. Suppose perturbation 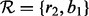 is given. Following the step 2, 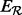 is the subset with only one mode *EM*_2_ because 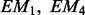 and *EM*_5_ contain reaction *b*_1_ and *EM*_1_ and *EM*_3_ contain both *r*_2_. Then we only refer to 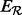 to compute the impacted reaction set as step 3. In this example, the impacted reactions are 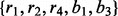 and the FBID of 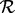 is 5.

### 2.3 Linear programming–based computation

Because the reduced feasible set 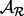 is defined in (3) by linear constraints, we propose an alternative algorithm to the EM-based approach based on linear programming (LP) to compute the FBID of a perturbation. Given a perturbation 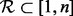 and a reaction 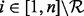, we consider the following optimization problems to decide whether reaction *i* is impacted by 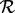:

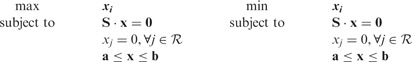


In other words, we perform FVA following each gene knock-out. However, contrary to classical use of FVA (Shlomi *et al.*, 2009), we are just interested here in assessing whether the solutions to both optimization problems are 0 or not. Indeed, it is easy to see that reaction *i* is impacted by perturbation 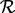 according to Definition 1 if and only if the solutions of both problems are equal to 0 because this means that in the feasible set of both problems, which is exactly 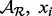 is constrained to be 0.

In practice, to compute the FBID of a perturbation 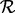 containing *K* reactions, one should solve a total of 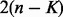 LP, corresponding to two problems for each reaction 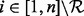. Because each LP can be solved in polynomial time, we obtain a polynomial time algorithm to compute the impact of all perturbations containing a bounded number of reactions. In addition, as all LP are related to each other, significant speed-up can be obtained by using warm restart, as implemented in the fastFVA software (Gudmundsson and Thiele, 2010). Further speed-up is also possible by solving batches of LP in parallel on a distributed computing environment.

### 2.4 Implementation

We used both fastFVA (Gudmundsson and Thiele, 2010) and ILOG CPLEX (version 11.2) (http://www.ilog.com/products/cplex) to solve the LP instances of the LP-based method, and CellNetAnalyzer which is a free software running under MATLAB to compute the EMs of a network (Klamt *et al.*, 2007). All computations were performed on a PC with a Xeon CPU 3.33 GHz and 10 GB RAM running under the LINUX OS.

## 3 DATA

### 3.1 The *E.coli* metabolic networks

We use three versions of the *E.coli* metabolic network, as summarized in Table 1. The central network is from the KEGG database (Kanehisa and Goto, 2000; Kanehisa *et al.*, 2012), and iJE660 and iJO1366 are from the BiGG database (Orth *et al.*, 2011; Schellenberger *et al.*, 2010), stored as METATOOL and SBML formats, respectively. iJO1366 is the latest version of *E.coli* network, while we keep the older iJE660 in our experiments to allow comparison with previous work (Edwards and Palsson, 2000a,b; Reed *et al.*, 2003).

**Table 1.** The *E.coli* network with different versions

Versions	# Reactions	# Metabolites	# Genes
Central network	63	59	85
iJE660	627	438	660
iJO1366	2251	1136	1366

We should notice that these networks are obtained as closed systems, and thus, additional information like sources and biomass synthetics is needed to make these systems open (Edwards and Palsson, 2000a; Reed *et al.*, 2003; Wunderlich and Mirny, 2006). Sources provide compounds to be consumed, while biomass synthetics are compounds exhausted by the networks. In the implementation, we use two types of sources, detailed in the Supplementary Materials. The first source represents a minimal medium consisting mainly of energy source, carbon dioxide and oxygen. The other source is a rich environment, which covers the minimal medium together with 20 amino acids, biotin, thiamin and riboflavin, etc. The *E.coli* output biomass is given also in the Supplementary Materials.

### 3.2 Phenotypic data

To compare our *in silico* impact predictions with experimental data, we consider five datasets used in previous studies to assess the phenotypic consequences of gene knock-out.

The first dataset, collected from literature by Edwards and Palsson (2000a), measures the growth capability of 79 gene deletion mutants, among which 41 are essential, 36 are non-essential and 2 have been observed as either essential or non-essential. Following Wunderlich and Mirny (2006), we consider the predictions of any method on the later 2 genes as always correct to compute the accuracy of the prediction, while we remove them to compute receiver operating characteristic (ROC) curves.

The second dataset (*insertional mutants*), collected by Badarinarayana *et al.* (2001) and further used by Wunderlich and Mirny (2006), gives the growth rate of 481 mutants obtained by knock-out of single genes, among which 222 with >50% decrease in growth rate are considered essential. While all genes are available in the iJE660 network, only 461 (including 218 essentials) are present in the iJO1366 network.

The third dataset is the combination of the first two ones. Although they contain genes in common, we follow Wunderlich and Mirny (2006) and consider them all different because they are part of different networks specific to each dataset.

The fourth dataset, collected by Gerdes *et al.* (2003) and further used by Wunderlich and Mirny (2006), evaluates the gene variability of 598 mutants, among which 120 are considered essential. While all genes are available in the iJE660 network, only 571 (including 117 essentials) are present in the iJO1366 network.

The fifth dataset is the KEIO collection, collected by Baba *et al.* (2006), which partitions 4288 mutants into 317 (including 14 newly added by Yamamoto *et al.*, 2009) essential and 3971 nonessential genes. Among them, 81 (respectively 144) essential and 554 (respectively 1222) nonessential genes are present in the iJE660 (respectively iJO1366) model.

Because these experimental datasets are under different conditions, we used different input sources and output biomass in the networks for the different datasets, as listed in Supplementary Files. In short, for the mutants collected from literature, the minimal medium set is used to reconstruct four distinct networks, each of which includes only one of the energy sources. For the insertional mutants, we reconstruct the network by adding the minimal medium input with all energy sources. We use the rich source set when analyzing the Gerdes dataset and KEIO collection. As for outputs, all analyses with iJE660 share the same biomass output (Supplementary Table S3), while for iJO1366, we use the core growth biomass proposed by Orth *et al.* (2011).

## 4 RESULTS

For each of the three *E.coli* metabolic networks listed in Table 1, we computed the FBID of each single gene deletion. Note that because a gene can catalyze several reactions, the perturbation set 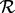 associated to a gene deletion is the set of reactions catalyzed by the gene. We first assess the computational performance of the approach, before assessing the ability of FBID to predict phenotypes and compare it with state-of-the-art methods.

### 4.1 Computation time

We proposed two algorithms to compute the FBID of a perturbation: one approach based on enumerating EM (Section 2.2), and one approach based on an LP formulation (Section 2.3). Table 2 shows the total computation time to perform the experiment on each network. For the LP-based approach, this is the total time to solve all LP with fastFVA; for the EM-based method, this is the time to compute the EMs of each network once with CellNetAnalyzer, and then output the list of impacted reactions for each gene deletion.

**Table 2.** Computational time for FBID computation

Versions	Computational time (s)	
	LP-based	EM-based
Central network	8	4
iJE660	252	> 7 days
iJO1366	10 234	> 7 days

We see that the EM-based method is fast for a small network but not efficient for large ones; in fact, CellNetAnalyzer did not manage to compute the EMs of both large networks within a week. This is coherent with the exponential complexity of the method. On the other hand, although many LP instances need to be solved for the LP-based method, we see that its polynomial complexity allows it to better scale to large networks. fastFVA (Gudmundsson and Thiele, 2010) manages to finish all computations on the largest network with 2251 reactions within ∼3 h, and is roughly two orders of magnitude faster than a naive implementation solving all LP instances independently from each other with CPLEX (see Supplementary Information).

Based on these observations, in what follows we only run the LP-based implementation with fastFVA to compute the FBIDs corresponding to the different genes and networks investigated for phenotypic prediction. The total computation times for both *E.coli* global metabolic networks (iJE660 and iJO1366) are summarized in the Supplementary Materials.

### 4.2 Phenotypic prediction

**Fig. 2.**
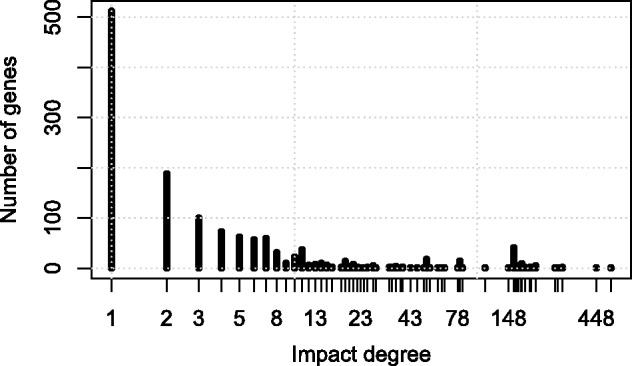
FBID distribution for the 1366 genes of the KEIO collection dataset computed on the iJO1366 metabolic network

The FBIDs computed on each network vary significantly between different genes. For example, Figure 2 shows the distribution of FBIDs for the 1366 genes of the KEIO collection dataset estimated on the iJO1366 model. While >80% of all genes have an FBID smaller than 10, it increases to 540 for the *msbA* (b0914) gene, a bacterial lipid flippase whose knock-out blocks ATP synthesis by oxidative phosphorylation, or 448 for *acpP* (b1094), a acyl carrier protein that catalyzes polyketide biosynthesis of holo-ACPS; unsurprisingly, both are essential genes.

**Fig. 3.**
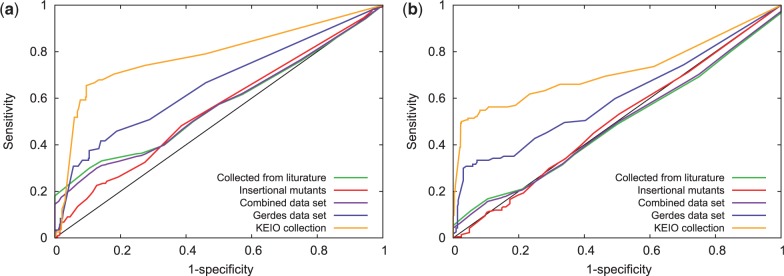
ROC curves for phenotype prediction from the FBID on various datasets, using both the iJE660 metabolic network (left) and the larger iJO1366 network (right)

To assess more quantitatively how predictive the FBID is for gene essentiality, we systematically compared the FBID corresponding to each gene deletion with the corresponding experimental phenotypic data, for both versions of the large *E.coli* metabolic network (iJE660 and iJO1366). In each experimental data, the genes are separated in two classes corresponding to genes with a large or small phenotypic impact. By thresholding the FBID to some level, we can predict that genes with an FBID above the threshold should have large phenotypic impact, while those below the threshold should not. Figure 3 shows the ROC curve for each dataset and each network, corresponding to the sensitivity plotted as a function of 1-specificity when we vary the FBID threshold. In addition, we show on Table 3 the area under the ROC curve (AUC) and the accuracy reached in each case, when the FBID threshold is set for each phenotypic dataset to maximize the accuracy as in Wunderlich and Mirny (2006).

**Table 3.** Performance of FBID on gene essentiality prediction, using both iJE660 and iJO1366

Experimental data	AUC		Accuracy	
	iJE660	iJO1366	iJE660 (%)	iJO1366 (%)
Collected from literature	0.57	0.49	68	63
Insertional mutants	0.55	0.50	57	52
Combined dataset	0.57	0.49	59	54
Gerdes dataset	0.66	0.60	82	83
KEIO collection	0.78	0.72	89	93

We can see that phenotype prediction for the iJE660 and iJO1366 *E.coli* networks are overall similar, with an advantage for the former on all phenotype datasets. The performance on the first dataset (collected from literature) is rather disappointing. This can be explained, to some extent, by the fact that we had to modify the network by using the minimal inputs together with distinct carbon sources, which resulted in many metabolites and reactions being always inactive at steady state. The performance on the insertional mutants dataset is also not good and may also be due in part to the particular context of using minimal inputs. For the Gerdes dataset and KEIO collection, FBID performs pretty well on both networks, reaching an AUC of 0.66 and 0.78 for iJE660 and 0.6 and 0.72 for iJO1366, respectively.

To compare the performance of FBID with existing approaches, we first focus on the iJE660 *E.coli* network that was used by Wunderlich and Mirny (2006) to compare SA (Wunderlich and Mirny, 2006), FBA (Edwards and Palsson, 2000b), MOMA (Segre *et al.*, 2002) and EMA (Stelling *et al.*, 2002). Results are summarized in Table 4, where we directly report the accuracies provided by Wunderlich and Mirny (2006) for existing methods.

**Table 4.** Comparison of the accuracy of FBID with different methods using the iJE660 network

Experimental data	Method				
	FBID (%)	SA (%)	FBA (%)	MOMA (%)	EMA (%)
Collected from literature	68	71	86	—	90
Insertional mutants	57	60	58	59	—
Combined dataset	59	62	62	—	—
Gerdes dataset	82	74	82	—	—

On the mutants collected from literature, our approach based on FBID is clearly worse than SA, FBA and EMA, which reach high accuracy (90% for EMA). This can be explained, to some extent, because this collection includes genes that only catalyze the central metabolism (Edwards and Palsson, 2000a) where alternative paths are numerous when we block a single gene. Therefore, although changes in optimal fluxes captured by FBA, or decrease in number of EMs captured by EMA, correlate well with growth rate, our approach meets difficulties in finding important fluctuations in the number of reactions that become completely inhibited when a gene is deleted. On the insertional mutant dataset, all methods reach a similar level of accuracy, with a slight advantage for SA and MOMA over FBA and FBID. On the larger Gerdes dataset, FBID and FBA reach the same level, and clearly outperform SA.

**Fig. 4.**
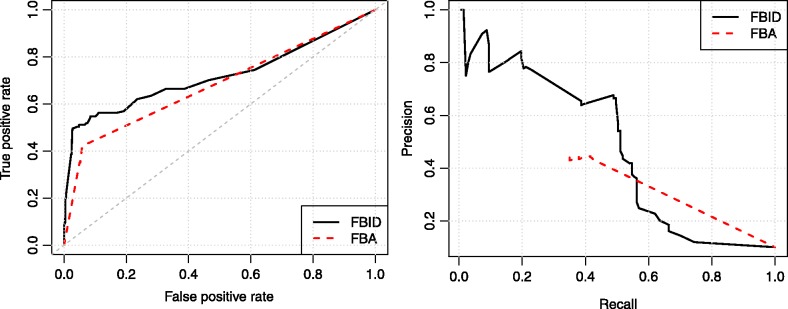
ROC curve (left) and precision-recall curve (right) for phenotype prediction with FBID and FBA on the Keio dataset using the iJO1366 network

To further investigate the performance of FBID on large networks, we compare it with FBA on the largest iJO1366 network for the prediction of gene essentiality as defined in the KEIO collection. Figure 4 shows the ROC and precision-recall curves of both methods. We see that FBID (AUC = 0.72, accuracy = 93%) outperforms FBA (AUC = 0.68, accuracy = 89%) on this experiment, confirming the potential of FBID on large networks.

**Fig. 5.**
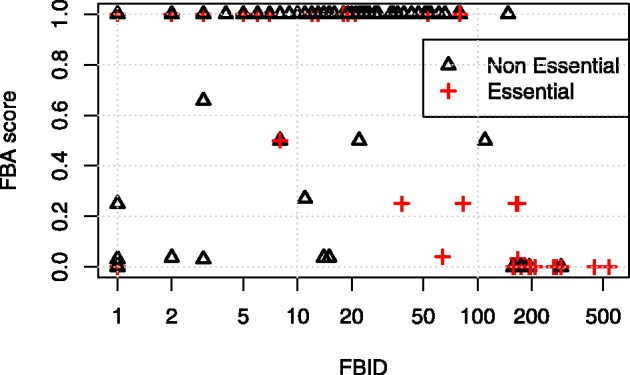
FBID and FBA scores for all genes in the Keio dataset analyzed with the iJO1366 network. Crosses correspond to experimentally essential genes

As shown on Figure 5, the predictions of FBID and FBA are correlated: genes with a large FBID (on the right) often have a small FBA score (near the bottom), corresponding to two notions of essentiality. However, the correlation is not perfect, and we observe, for example, a number of non-essential genes with a small FBA score and a small FBID (near the bottom left); in that case, the FBID is a better indicator of essentiality. Another advantage of FBID over FBA is the fact that FBA has difficulties to make a difference between the genes predicted to be essential. For example, 109 genes out of 1322 have a minimum FBA score of 0, corresponding to a complete blockage of fluxes; however, only 48 of them (44%) are truly essential. This means that FBA can not predict essentiality with >44% precision, as can be seen on the precision-recall curve (Fig. 4). On the contrary, FBID is better able to rank the genes with large scores, and can reach much higher precision than FBA near the top of the list. This is particularly relevant for applications where we want to predict a few essential genes with high precision. More details about FBA and FBID essentiality prediction can be found in the Supplementary Information.

## 5 DISCUSSION

We have proposed FBID, a new definition of impact degree, which can not only efficiently deal with the reversible reactions in metabolic networks but also have the state conditions being taken into account. To compute the FBID against perturbations, we proposed two algorithms, an LP-based method and an EM-based algorithm. The advantage of the LP-based method is that it can solve all LP instances individually, can strongly benefit from warm restart techniques and is amenable to parallelization. Contrary to other LP-based formalisms like FBA, FVA or MOMA, it does not depend on a subjective definition of a relevant objective function. Although computational cost of the LP-based method grows with the network size and the number of perturbations to be tested, the overall time complexity is still bounded polynomially. If we are interested in only a few candidate perturbations, then only the corresponding LP need to be solved. The EM-based method, on the other hand, can compute the FBID of specific perturbations fast for middle-scale networks. The main computational advantage of this approach is that the computation of EMs needs to be performed only once, no matter how many perturbation we want to test—including perturbations involving several reactions. This advantage vanishes for large-scale metabolic networks, however, because of the exponential complexity of computing EMs and the lack of efficient algorithms for that purpose.

We carried out computational experiments by using *E.coli* metabolic networks. The results on computational time for calculating the FBID of different sized networks show that the LP-based method implemented with fastFVA is efficient, while the EM-based method did not return any result for large networks owing to the difficulty of computing the EMs. In terms of phenotype prediction, we obtained poor results when we tested metabolic networks with a minimal source input because many metabolic paths are always closed in this case. Comparison of the performance of phenotype prediction with some existing methods indicates that the FBID performs as well as other models or even better, particularly on large networks.

The interpretation we give of the FBID in terms of EMs makes an interesting link with existing EM-based methods that measure how many EMs disappear when we inhibit a reaction (Behre *et al.*, 2008; Wilhelm *et al.*, 2004). In our case, we also enumerate the list of EMs that remain once the reaction is inhibited, but instead of focusing on the *number of EMs* remaining, we focus instead on the *number of reactions* that can still be activated in the remaining EMs. Although the number of EMs in a network has been used as a measure of flexibility and as an estimate of fault-tolerance (Stelling *et al.*, 2002), we propose here that the amount of reactions inactivated in cascade may be a better indicator of gene essentiality. Of course, the *number* of reactions inactivated is itself a crude measure, and investigating variants such as weighting reactions by their ‘importance’ before counting them may be interesting future work.

## Supplementary Material

Supplementary Data
