# Supplementary material for: Flux balance impact degree: a new definition of impact degree to properly treat reversible reactions in metabolic networks
Source: Bioinformatics. 2013 Jul 10;29(17):2178–85. doi: 10.1093/bioinformatics/btt364 (PMC3740629; doi:10.1093/bioinformatics/btt364)
Supplement: Supplementary Data [file supp_29_17_2178__index.html]

Flux balance impact degree: A new definition of impact degree to properly treat reversible reactions in metabolic networks — Flux balance impact degree: a new definition of impact degree to properly treat reversible reactions in metabolic networks — Flux balance impact degree: a new definition of impact degree to properly treat reversible reactions in metabolic networks — Supplementary Data 

# Flux balance impact degree: a new definition of impact degree to properly treat reversible reactions in metabolic networks

## 

files

**Files in this Data Supplement:**

- Supplementary Data - xlsx file
